# Inhibition of iNOS activity enhances the anti-tumor effects of alpha-galactosylceramide in established murine cancer model

**DOI:** 10.18632/oncotarget.6172

**Published:** 2015-10-19

**Authors:** Hiroyasu Ito, Tatsuya Ando, Mitsuru Seishima

**Affiliations:** ^1^ Department of Informative Clinical Medicine, Gifu University Graduate School of Medicine, Yanagido, Gifu, Japan

**Keywords:** cancer immunotherapy, alpha-garactosylceramide, induced nitric oxide synthase, tumor antigen-specific immune response, MDSC

## Abstract

Alpha-garactosylceramide (GalCer) has been shown to have anti-tumor effect in the basic research and clinical studies. However, anti-tumor effect of GalCer is limited. The administration of GalCer increases the production of IFN-γ which is involved in the suppression of tumor growth. On the other hand, the enhancement of IFN-γ production increases immunosuppressive factors such as nitric oxide. This suppressive action might impair the anti-tumor effect of GalCer. In the present study, we evaluated the anti-tumor effect of GalCer in the absence of inducible nitric oxide synthase (iNOS). In lung metastatic model, the number of tumor nodules in the lung of iNOS-KO mice treated with GalCer was significantly reduced compared with that of WT mice treated with GalCer. Moreover, L-NAME, which is the inhibitor for iNOS, enhanced the anti-tumor effect of GalCer in lung metastatic model. The frequency of CD8+ cells in bronchoalveolar lavage fluid increased in iNOS-KO mice treated with GalCer. The administration of GalCer increased the frequency of myeloid-derived suppressor cells (MDSCs) in the lung from tumor-bearing WT mice, but the increase of MDSCs in the lung was not induced in iNOS-KO mice. The subcutaneous tumor experiments revealed that the administration of GalCer in the absence of iNOS expression significantly enhanced the induction of tumor antigen-specific response. Finally, our results indicated that the inhibition of iNOS expression could enhance the therapeutic efficacy of GalCer via the increase of tumor antigen-specific immune response and the suppression of MDSCs.

## INTRODUCTION

Alpha-galactosylceramide (GalCer) is identified as the ligand of Vα14+ natural killer (NK) T cells. Vα14+ NKT cells distinct from mainstream T cells, B cells and NK cells have been identified. These cells are found in relative abundance in tissues such as spleen, bone marrow, thymus, and liver, and are characterized by the co-expression of NK cell receptors and invariant T cell receptors encoded by Vα14 and Jα18 gene segments [1]. Many reports previously demonstrated that the administration with GalCer induces the anti-tumor activity *via* the activation of NKT cells. The activated NKT cells can secrete various cytokines, and these cytokines contribute to the GalCer-induced anti-tumor effect *in vivo* [2–6]. However, the administration with GalCer alone is not so effective. Therefore, several reports evaluated the anti-tumor effect of GalCer by the combination with IL-12 or IL-18 [7, 8].

Inducible nitric oxide synthase (iNOS) is an enzyme that produces nitric oxide (NO) in several situations. In particular, NO promotes angiogenesis, metastasis, and immunosuppression in tumor microenvironment [9]. Various tumor cells can induce NO production *via* the up-regulation of iNOS expression, and iNOS expression is involved in the prognosis of the patient with any cancer [10, 11]. Previous studies demonstrated that myeloid-derived suppressor cells (MDSCs) also produce NO and suppress the host immune response in tumor microenvironment [12]. Thus, NO production contributes to the progression of cancer and it might be critical to suppress the expression of iNOS for cancer immunotherapy. Recent reports examined that the administration of GalCer enhanced the iNOS expression in EAE model [13]. The co-administration with GalCer and toll like receptor (TLR) agonist extremely enhanced NO production [14, 15]. Thus, the activation of NKT cells is effective for anti-tumor immunity *via* various cytokines, but is counteracted by the simultaneous induction of iNOS which has immunosuppressive effect in tumor-bearing animals. In the present study, we addressed the hypothesis that the inhibition of iNOS activity during cancer therapy using GalCer will enhance the tumor antigen-specific host immune response to inhibit tumor growth. We were able to show that the administration of GalCer and simultaneous inhibition of iNOS activity promote the tumor antigen-specific immune response, leading to the suppression of established lung metastasis and subcutaneous tumor model *in vivo*.

## RESULTS

### Up-regulation of iNOS expression after the administration with GalCer

**Figure 1:**
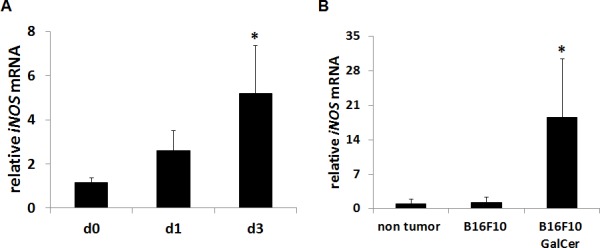
Up-regulation of iNOS expression after GalCer administration in tumor-bearing mice B16F10 cells (3 × 10^5^/mouse) were intravenously administered to WT mice. WT mice were intraperitoneally injected with GalCer (2 μg/mouse) at 7 days after the inoculation of tumor cells. **A.** The relative expression levels of iNOS mRNA in the lung of tumor-bearing mice treated with GalCer were measured by real-time RT-PCR. **B.** CD11b+ cells were magnetically isolated from BALF, and iNOS mRNA expression of CD11b+ cells were measured by real-time RT-PCR. The results were normalized to the expression of 18S rRNA. Each value is shown as mean and SEM for three mice. * indicates statistically significant differences.

GalCer have been recently used for cancer therapy in basic and clinical research. Although the administration with GalCer enhances the host immune response, immunosuppressive factors, including iNOS, are simultaneously induced by GalCer. We first examined the iNOS expression in lung of B16 F10 cells-bearing mice after intraperitoneal injection with GalCer. As shown in Figure 1A, iNOS mRNA expression was increased in the lung of tumor-bearing WT mice after GalCer injection (*P* < 0.05). We next examined the mRNA expression of iNOS in CD11b+ cells of tumor-bearing WT mice (Figure 1B). CD11b+ cells were magnetically collected from bronchoalveolar lavage fluid (BALF) of tumor-bearing mice by MACS system. The iNOS mRNA expression of CD11b+ cells in BALF was extremely up-regulated by the administration with GalCer (*P* < 0.05).

### Anti-tumor effect of GalCer was enhanced by the suppression of iNOS activity in lung metastasis models

**Figure 2:**
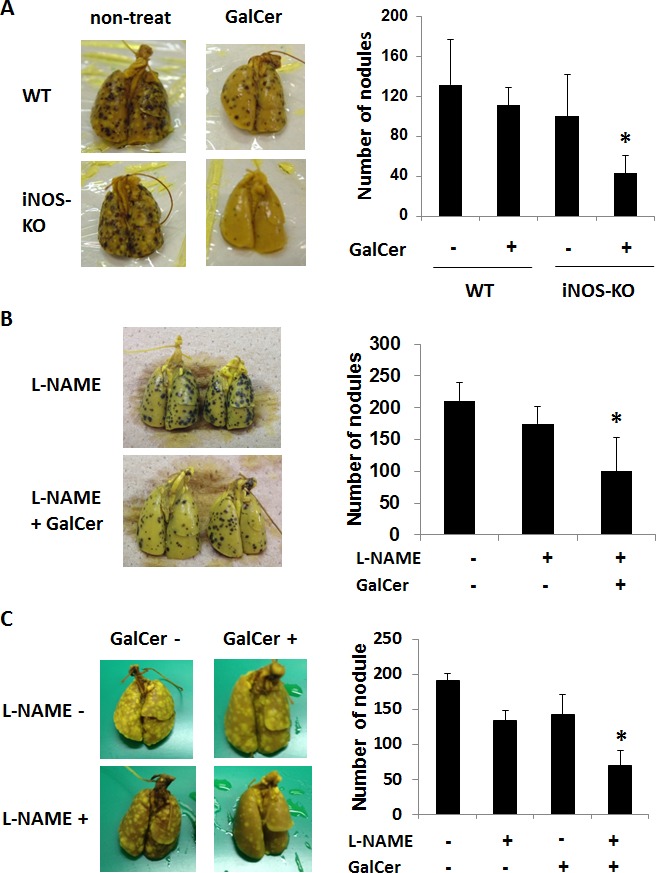
Inhibition of iNOS expression enhanced the anti-tumor effect of GalCer on lung metastatic cancer model **A.** B16F10 cells (3 × 10^5^/mouse) were intravenously administered to WT and iNOS-KO mice on day 0. Tumor-bearing mice were intraperitoneally injected with GalCer (2 μg/mouse) at 7 days after the inoculation of tumor cells. Mice were killed at 7 days after the injection of GalCer, and their lungs were removed to count superficial metastatic nodules. The number of superficial metastatic lung tumor was significantly decreased in iNOS-KO mice treated with GalCer (*P* < 0.05). **B.** B16F10 cells (3 × 10^5^/mouse) were intravenously administered to WT mice on day 0. WT mice were administered with L-NAME orally at 2 mg/ml in drinking water for 1 days before and 7 days after GalCer injection. Mice were killed at 7 days after the injection of GalCer. The number of superficial metastatic lung tumor was significantly reduced in mice with L-NAME and GalCer (*P* < 0.05). **C.** CT26 cells (2 × 10^5^/mouse) were intravenously administered to WT mice on day 0. The mice were orally administered L-NAME in drinking water at 6 days after tumor injection, and intraperitoneally injected with GalCer 1 day later. Mice were killed at 7 days after the injection of GalCer. The number of superficial metastatic lung tumor was significantly reduced in mice with L-NAME and GalCer (*P* < 0.05). Each value is shown as mean and SEM for 4-6 mice.

B16F10 cells and C26 cells were intravenously injected to WT or iNOS-KO mice to establish lung metastasis models. Tumor nodules in the lung could be confirmed at 5 days after the administration with tumor cells (data not shown). GalCer (2 μg/mouse) was intraperitoneally administered into B16F10 cells-bearing WT and iNOS-KO mice 7 days after the inoculation of tumor cells. Seven days after GalCer injection, mice were killed, and their lungs were removed to count superficial metastatic nodules (Figure 2A). The number of nodules in the lung was significantly reduced in iNOS-KO mice treated with GalCer (*P* < 0.05). N^G^-nitro-L-arginine methyl ester (L-NAME) is an iNOS inhibitor; hence, we used this agent to substantiate data obtained from iNOS-KO mice. WT mice were orally administered with L-NAME at 0 or 2 mg/ml in drinking water for 1 day before GalCer injection. The co-administration with GalCer and L-NAME significantly decreased the number of tumor nodules in the lung (*P* < 0.05) (Figure 2B). Next, we examined the effect of GalCer and L-NAME on the different lung tumor model. C26 cells were inoculated to WT mice, and GalCer and L-NAME were administered into C26 tumor-bearing mice. The co-administration with GalCer and L-NAME significantly reduced the number of tumor nodules in C26 lung tumor model (*P* < 0.05) (Figure 2C).

### Effect of GalCer treatment on the phenotype of lymphocyte in BALF from tumor-bearing WT and iNOS-KO mice

**Figure 3:**
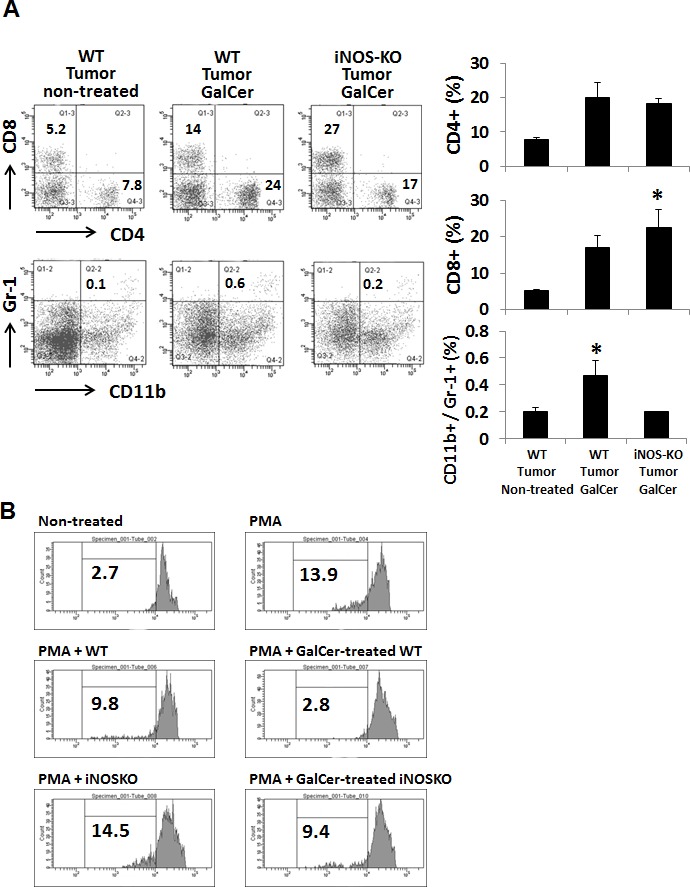
Changes of the proportion of CD8+ and CD11b+/Ly6G+ cells in BALF cells from tumor-bearing WT and iNOS-KO mice treated with GalCer The lymphocytes in BALF from tumor-bearing WT and iNOS-KO mice were isolated 3 days after GalCer administration. **A.** Data show the percentage of CD4+, CD8+, and CD11b+/Ly6G+ cells. Each data point and error bar represent the mean and SEM, respectively, of data from triplicate samples. * indicates statistically significant differences. Closed bar; WT mice, open bar; iNOS-KO mice. **B.** BALF cells were isolated from WT and iNOS-KO mice administered with GalCer. Naïve splenocytes labeled with CFSE were co-cultured with BALF cells in 96-well plates at a cell-count ration of 1/1 for 3 days in the presence of PMA. Data were representative of at least three independent experiments with similar result.

We next measured the frequency of CD4+, CD8+, and CD11b+/Ly6G+ cells in BALF of tumor-bearing WT and iNOS-KO mice at 3 day after GalCer administration. As shown in Figure 3A, the frequency of CD8+ cells was significantly increased in iNOS-KO mice treated with GalCer. On the other hand, the rate of CD11b+/Ly6G+ cells in iNOS-KO mice treated with GalCer was decreased compared to that in WT mice treated with GalCer. There was no difference between WT and iNOS-KO mice treated with GalCer in the ratio of CD4+ cells. Moreover, CFSE-based T cell suppression assay revealed that the proliferation of CFSE-labeled CD3+ cells co-cultured with BALF cells from GalCer-treated WT mice was more suppressed compared with those from non-treated WT mice or GalCer-treated iNOS mice (Figure 3B).

### IFN-γ, FasL, CCL2, and CXCL9 mRNA expression in the lung of tumor-bearing WT and IDO-KO mice treated with GalCer

**Figure 4:**
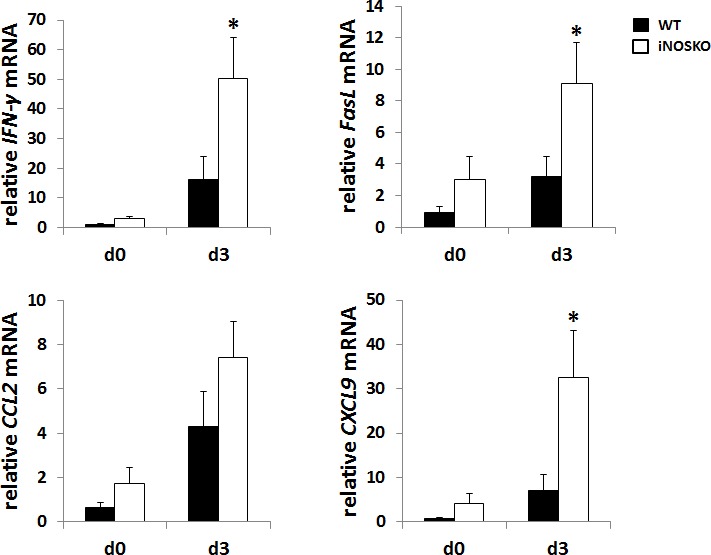
The mRNA expression of IFN-γ, FasL, and CXCL9 in the lung from tumor-bearing iNOS-KO mice was up-regulated after GalCer administration B16F10 cells (3 × 10^5^/mouse) were intravenously administered to WT mice on day 0. WT mice were intraperitoneally injected with GalCer at day 7 after the inoculation of tumor cells. The relative expression levels of IFN-γ, FasL, CCL2, and CXCL9 mRNA in the lung of WT and iNOS-KO mice treated with GalCer were measured using real-time RT-PCR. The results were normalized to the expression of 18S rRNA. Each data point and error bar represent the mean and SEM, respectively, of data from triplicate samples. * indicates statistically significant differences.

IFN-γ and FasL are critical on the establishment of anti-tumor immunity. Moreover, CCL2 and CXCL9 are involved in the chemoattractant of T cells [16, 17]. Next, we measured mRNA levels for IFN-γ, FasL, CCL2, and CXCL9 in the lung from tumor-bearing WT and iNOS-KO mice after GalCer administration (Figure 4). The mRNA expression of IFN-γ, FasL, and CXCL9 significantly increased in iNOS-KO mice at 3 days after GalCer administration. Although the expression of CCL2 mRNA was up-regulated in WT and iNOS-KO mice after the administration with GalCer, there was no difference between WT and iNOS-KO mice in the expression of CCL2 mRNA after GalCer injection (Figure 4).

### Effect of GalCer injection on subcutaneous primary tumors in WT and iNOS-KO mice

**Figure 5:**
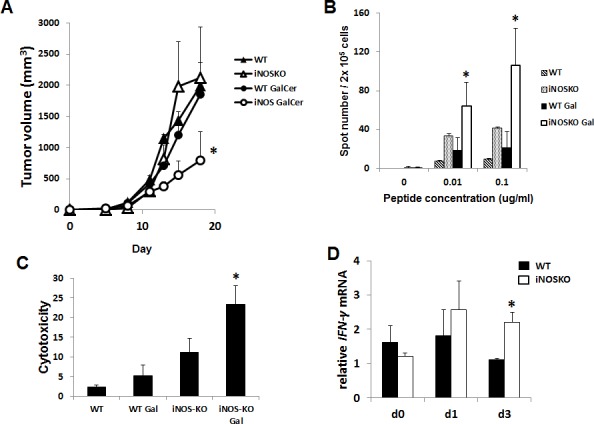
Inhibition of iNOS expression enhanced the anti-tumor effect of GalCer in a subcutaneous tumor model EG7 cells (1 × 10^6^/mouse) were subcutaneously inoculated into the flank of WT and iNOS-KO mice on day 0. After the tumors became palpable, the mice were intraperitoneally injected with GalCer (2 μg/mouse). **A.** Data show the mean ± SEM increase in tumor size 6-8 mice per group, from two experiments. **B.** DLN in tumor-bearing WT and iNOS-KO mice were isolated 7 days after GalCer intraperitoneal injection. These DLN cells were stimulated with OVA peptide in vitro and monitored for IFN-γ production using ELISPOT assay. Results represent the mean ± SEM of 4 mice per group. The number of spot was significantly increased in DLN cells from iNOS-KO mice treated with GalCer (*P* < 0.05). **C.** Isolated effector cells (CD8+ T cells) from DLN of WT and iNOS-KO mice treated with GalCer were incubated for 6 h with CFSE-labeled EG7 at an effector to target cell ratio of 20 to 1. Each data point and error bar represent the mean and SE, respectively, of results for triplicate samples. **D.** The relative expression of IFN-γ mRNA in DLN of WT and iNOS-KO mice treated with GalCer was measured using real-time RT-PCR. The results were normalized to the expression of 18S rRNA. The expression of IFN-γ mRNA was significantly increased in iNOS-KO mice treated with GalCer (*P* < 0.05).

**Figure 6:**
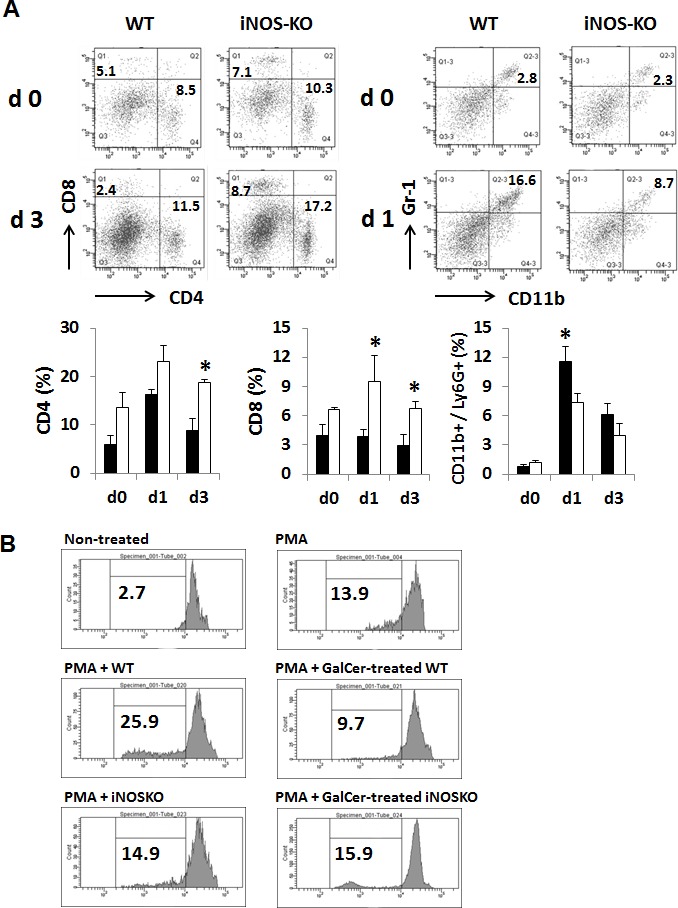
Changes of the proportion of CD8+ and CD11b+/Ly6G+ cells in the tumor infiltrating lymphocytes from tumor-bearing WT and iNOS-KO mice treated with GalCer The tumor infiltrating lymphocytes from tumor-bearing WT and iNOS-KO mice were isolated 1 and 3 days after GalCer administration. **A.** Data show the percentage of CD4+, CD8+, and CD11b+/Ly6G+ cells. Each data point and error bar represents the mean and SEM, respectively, of data from triplicate samples. * indicates statistically significant differences. Closed bar; WT mice, open bar; iNOS-KO mice. **B.** Splenic CD11b+ cells were isolated from WT and iNOS-KO mice administered with GalCer. Naïve splenocytes labeled with CFSE were co-cultured with CD11b+ cells in 96-well plates at a cell-count ration of 1/1 for 3 days in the presence of PMA. Data were representative of at least three independent experiments with similar result.

We next examined the anti-tumor effect of GalCer in WT and iNOS-KO mice using subcutaneous tumor models. EG7 cells were inoculated in to the right flank of WT and iNOS-KO mice, and GalCer was intraperitoneally injected when the tumor size grew to approximately 5 mm in diameter. The administration with GalCer significantly impaired the growth of subcutaneous tumor in iNOS-KO mice (*P* < 0.05) (Figure 5A). To clarify the mechanism by which GalCer injection promotes anti-tumor effect in iNOS-KO mice, cells were isolated from tumor DLN of mice bearing subcutaneous EG7 tumor. The DLN cells were cultured *ex vivo* with the OVA SIINFEKL peptide, which is a CD8-restricted epitope expressed by EG7. DLN cells from tumor-bearing WT and iNOS-KO mice treated with GalCer responded to stimulation by OVA peptide by secreting IFN-γ. As shown in Figure 5B, the number of spot significantly increased by the stimulation with OVA peptide in DLN cells from iNOS-KO mice treated with GalCer (*P* < 0.05). Moreover, we examined the EG7-specific lysis caused by CD8+ T cells in DLN cells from WT and iNOS-KO mice treated with GalCer *ex vivo* (Figure 5c). Specific cytotoxicity of CD8+ T cells to EG7 cells in DLN cells from the iNOS-KO mice treated with GalCer was significantly increased compared to that from WT mice treated with GalCer (*P* < 0.05). We measured the expression of IFN-γ mRNA in DLN of WT and iNOS-KO mice at 3 days after GalCer administration (Figure 5D). The expression of IFN-γ mRNA in iNOS-KO mice was significantly increased after GalCer administration compared with that in WT mice. In the tumor, the frequency of CD8+ cells in iNOS-KO mice was significantly increased after GalCer administration compared to that in WT mice (*P* < 0.05) (Figure 6A). On the other hand, the frequency of CD11b+/Ly6G+ cells in the tumor of iNOS-KO mice treated with GalCer decreased compared to that of WT mice (*P* < 0.05). In CFSE-based T cell suppression assay, the proliferation of CFSE-labeled CD3+ cells co-culture with CD11b+ cells from GalCer-treated WT mice was significantly suppressed compared with those from non-treated WT mice and GalCer-treated iNOS mice (Figure 6B).

## DISCUSSION

In the present study, we have shown the inhibition of iNOS activity significantly enhanced the anti-tumor effect of GalCer in lung metastatic tumor model and primary subcutaneous tumor model. The administration with GalCer enhanced the tumor antigen-specific response and cytotoxic activity in the absence of iNOS expression. Moreover, the increase of CD8+ cells and the decrease of CD11b+/Ly6G+ cells within tumor might be involved in the impairment of tumor growth.

GalCer enhances host immune system *via* NKT cell activation. The activation of NKT cells induces the amount of Th1 cytokines, including IFN-γ and Th2 cytokines. Many reports indicated that the increase of Th1 cytokines production lead to the induction of anti-tumor immunity [18]. Therefore, the anti-tumor effect of GalCer was recently evaluated in many animal and clinical studies [19–21]. It is well-known that IFN-γ is a strong inducer of iNOS. A recent report demonstrated that the administration with GalCer also induces the expression of iNOS and enhanced the NO production [22]. Indeed, the injection of GalCer in tumor-bearing mice up-regulated the mRNA expression of iNOS in the lung (Figure 1A). In particular, iNOS expression in CD11b+ cells was markedly increased after the administration with GalCer. Monoctye/macrophage expresses CD11b molecules on surface and induces iNOS by the stimulation of IFN-γ [23]. In the present study, the iNOS expression in CD11b+ cells of BALF might be enhanced by IFN-γ from activated NKT cells.

As shown in Figure 2, the inhibition of iNOS activity by genetically modification or the administration with L-NAME significantly enhanced the anti-tumor effect of GalCer in lung metastatic tumor model. These results indicated that the enhancement of anti-tumor response by GalCer was not dependent on the type of cancer cell line or mice. In subcutaneous tumor model, the anti-tumor effect of GalCer similarly increased in iNOS-KO mice (Figure 5A). The frequency of CD8+ cells in BALF of iNOS-KO mice was significantly increased by the administration with GalCer compared to that of WT mice (Figure 3A). In subcutaneous tumor model, CD8+ cells within tumor also increased in iNOS-KO mice after GalCer injection (Figure 6A). It is well-known that CD8+ cells have cytotoxic activity against tumor cells and are critical in cancer immunotherapy [24, 25]. The increase of CD8+ cells within tumor might lead to the enhancement of anti-tumor effect of GalCer in iNOS-KO mice. In general, CD8+ T cells are involved in anti-tumor immunity *via* the production of IFN-γ, FasL, and perforin [26, 27]. In the present study, the treatment of GalCer in iNOS-KO mice increased the expression of IFN-γ and FasL in the lung (Figure 4). These data supported that CD8+ T cells within tumor of tumor-bearing iNOS-KO mice increased after GalCer administration. The expression of CXCL9 mRNA was also enhanced in iNOS-KO mice treated with GalCer (Figure 4). CXCL9 is known as the T-cell chemoattractant, which is induced by IFN-γ. The up-regulation of CXCL9 in the lung might be involved in the increase of CD8+ cells in BALF of tumor-bearing iNOS-mice treated with GalCer. Moreover, we found that the frequency of CD11b+/Ly6G+ cells in iNOS-KO mice treated with GalCer was lower than that in WT mice treated with GalCer (Figures 3A and 6A). MDSCs are broadly defined as CD11b+/Ly6G+ cells. Several previous reports demonstrated that MDSCs were involved in the impairment of host immune systems at tumor-bearing animals [28, 29]. Recent studies demonstrated that iNOS enhanced the recruitment and induction of functional MDSCs [30, 31]. The present data also indicated that the frequency of MDSCs in the tumor was significantly decreased in iNOS-KO mice compared to that in WT mice. Moreover, CFSE-based proliferation assay revealed that BALF cells and CD11b+ cells from GalCer-treated WT mice had an ability to suppress the proliferation of T cells (Figures 3B and 6B). These suppressions were reduced in BALF cells and CD11b+ cells from GalCer-treated iNOS-KO mice. The absent of iNOS activity failed the induction of functional MDSCs after the treatment of GalCer. The decrease of functional MDSCs might be involved in the enhancement of anti-tumor effect in iNOS-KO mice treated with GalCer.

The inhibition of iNOS activity also enhanced the anti-tumor effect in subcutaneous tumor model (Figure 5A). ELISPOT assay revealed that tumor antigen-specific immune response was significantly increased in iNOS-KO mice treated with GalCer (Figure 5B). Moreover, CD8+ cells in DLN of GalCer-treated iNOS-KO mice extremely had the tumor specific cytotoxicity. The expression of IFN-γ in DLN also increased in iNOS-KO mice treated with GalCer (Figure 5C). These results indicated that the inhibition of iNOS expression enhanced the tumor antigen-specific immune response after the administration of GalCer. Recent study demonstrated that the inhibition of iNOS activity enhanced the anti-tumor effect of TLR7 agonist in subcutaneous tumor model [32]. Similarly, the anti-tumor effect of GalCer was enhanced by the inhibition of iNOS activity in subcutaneous tumor model. The enormous basic studies and clinical trials evaluated the anti-tumor effect in cancer immunotherapy. However, few anti-cancer therapies can completely eliminate the tumor in subcutaneous tumor model. Many studies used the agents which excessively stimulate host immune response like GalCer or TLR agonists. These agents can induce pro-inflammatory cytokines and IFNs in cancer immunotherapy. Previous reports demonstrated that these factors could induce IL-10, iNOS, and indoleamine 2,3 deoxgenase that have the immune suppressive effect [33, 34]. The induction of excessive host immune response promotes the immune suppressive factors in host. Therefore, the anti-tumor efficacy of monotherapy using immune stimulate agent might be reduced by the several suppressive factors induced by these agents.

In conclusion, the present study showed that the inhibition of iNOS significantly enhanced the anti-tumor effect of GalCer in lung tumor model and subcutaneous primary tumor model. The combination therapy of GalCer and iNOS inhibitor might be a new strategy for cancer immune therapy.

## MATERIALS AND METHODS

### Mice

Male C57BL/6J (H-2d) wild-type (WT) mice and BALB/c mice (age, 8-10 weeks; weight; 25-30 g) were obtained from Japan SLC Inc. (Shizuoka, Japan). iNOS knockout (KO) mice with a C57BL/6J background were obtained from Jackson Laboratory (Bar Harbor, ME). All procedures were conducted in accordance with the National Institutes of Health Guide for the Care and Use of Laboratory Animals, and with the guidelines for the care and use of animals established by the Animal Care and Use Committee of Gifu University.

### Cell lines and reagents

The B16-F10 melanoma cell line, colon carcinoma CT26 cells, EG7 cells used in this study were generously provided by Hidekazu Shirota (Laboratory of Experimental Immunology, Cancer and Inflammation Program, National Cancer Institute, Frederick, MD). These cells were cultured in suspension in RPMI 1640 (Invitrogen Ltd., Paisley, United Kingdom) containing 10% heat inactivated FCS (PAA Laboratories, GmbH, Linz, Austria), 2 mM L-glutamine, penicillin (100 units/ml), and streptomycin (100 g/ml). N^G^-nitro-l-arginine methyl ester (L-NAME) was purchased from SIGMA (St. Louis, MO). WT mice were administered with L-NAME orally at 2 mg/ml in drinking water for for 1 days before and 7 days after GalCer injection.

### *In vivo* tumor studies

Mice were intravenously injected with B16 F10 cells (3 × 10^5^ in C57BL/6 mice), and CT26 carcinoma cells (2 × 10^5^ CT26 carcinoma cells in BALB/c mice), and GalCer (2 μg/mouse) was administered into tumor-bearing mice 7 days after the inoculation of tumor cells. Seven days after GalCer injection, mice were killed, and their lungs were removed to count superficial metastatic nodules.

To establish the subcutaneous tumor model, mice were challenged with EG7-OVA cells (1 × 10^6^ cells in C57BL/6 mice). This tumor cell lines formed solid tumors when the tumor cells were subcutaneously inoculated into the flank. Tumor cells were implanted into the flank of WT or iNOS-KO mice on day 0. After the tumors became palpable (> 5 mm in diameter), the mice were intraperitoneally administered with GalCer (2 μg/mouse). Tumor growth curves were generated using 6-9 mice per group, and all results were derived by combining data from 2 to 3 independent experiments. As previous study, tumor size was calculated using the following formula: (length × width × height)/2.

### ELISPOT assay

The ELISPOT assay was performed as described previously [35]. Single-cell suspensions were prepared from the draining lymph node (DLN) of subcutaneous tumor-bearing mice treated with GalCer. A total of 2.0 × 10^5^ cells per well were stimulated for 14-16 h with 0, 0.1, or 1 μg/ml of ova SIINFEKL peptide in 96 well MultiScreen filter plates (Millipore, Billerica, MA) previously coated with monoclonal rat anti-IFN-γ antibody (Ab) (clone; R4-6A2) (BD Biosciences, San Jose, CA). The plates were washed and treated with biotinylated polyclonal goat anti-IFN-γ Ab (R&D Systems, Minneapolis, MN) followed by streptavidin alkaline phosphatase. Staining was visualized thorough the addition of a 5-bromo-4-chloro-3-indolyl phosphatase solution (Sigma-Aldrich, St. Louis, MO) and counted manually under 40× magnification. A single-blind reviewer counted the number of cytokine-secreting cells, and all data were generated by analyzing three separate wells per sample.

### Real-time reverse transcription-PCR

Total RNA was isolated from lung tissue, BALF cells and tumor tissue, and transcribed into complementary DNA (cDNA) using an RNeasy Mini Kit (QIAGEN, Hilden, Germany) and a high capacity cDNA transcription kit (Applied Biosystems, Foster City, CA). The resulting cDNA was used as a template for real-time RT-PCR along with primer-probe sets for iNOS, CCL2, CXCL9, FasL, IFN-γ, and 18S (TaqMan Gene Expression Assays; Applied Biosystems) and TaqMan universal PCR master mix (Applied Biosystems) according to the manufacturer's recommendations. 18S rRNA was used. Real-time RT-PCR was carried out using a Light-Cycler 480 system (Roche Diagnostic Systems, Basel, Switzerland). The calculation of relative gene expression differences was done by the comparative 2−ΔΔCT method.

### Flow cytometric analysis

BALF cells and intra-tumor lymphocytes were isolated from tumor-bearing mice treated with GalCer. Cell viability and number were assessed by trypan blue exclusion. For flow cytometry, 2 × 10^5^ cells were stained using a standard protocol. The following antibodies (Abs) were used: APC-labeled anti-mouse CD4 mAb (clone GK1.5; eBiosciences, San Diego, CA), PE-Cy7-labeled anti-mouse CD8 mAb (clone 53-6.7; eBiosciences, San Diego, CA), PE-Cy7-labeled anti-mouse CD11b mAb (clone M1/70; eBiosciences), and FITC-labeled anti-mouse Ly-6G mAb (clone RB6-8C5; BD Biosciences). Samples were acquired using a FACSCanto ll flow cytometer and data analysis was performed using FACSDiva software (BD Biosciences).

### Isolation of CD8+ and CD11b+ cells

CD8+ cells in DLN were isolated by magnetic beads conjugated with an anti-CD8 antibody (Miltenyi Biotec) as described in our previous report [36]. BALF cells were sorted by CD11b+ status using magnetic beads conjugated with an anti-CD11b antibody (Miltenyi Biotec). The magnetically labeled cells were purified using quadroMACS system (Miltenyi Biotec).

### Cytotoxicity assay

The cytolytic activity of EG7-specific lymphocytes was assessed using a fluorescent-based dye, 5- (and 6-) carboxyfluorescein diacetate succinimydyl ester (CFSE) as described previously [25]. Target EG7 cells were labeled with CFSE as follows: cells were suspended in PBS and concentrated to 1 × 106/ml. For targets cells, 0.5 μl of CFSE stock solution (5 mM) was added to 1 ml of cell suspension and incubated for 4 min at room temperature. For control targets cells, 0.5μl of diluted CFSE solution (100 μM) was used for labeling. Labeled targets and various numbers of effector cells were added to a final volume of 200 μl in each well of the 96-well round-bottom plates and incubated for 6 h at 37°C. After incubation, sensitive target cells were mixed with control cells in a tube with PBS containing 1% FCS and 0.1% sodium azide. The mixed cells were washed once and suspended in 4% paraformaldehyde-containing PBS and stored at 4°C in the dark until cytometer acquisition. Acquisition was performed using a FACSCantoII (BD Immunocytometry Systems). All samples were assayed in duplicate and the mean percentage of specific lysis was calculated as follows: % specific lysis = [(number of sensitive target cells in control sample − number of sensitive target cells in test sample) / number of sensitive target cells in control sample] × 100. The terms control sample and test sample indicate target cells incubated without added effector cells and target cells incubated with added effector cells, respectively.

### *In vitro* CFSE proliferation assay

BALF cells and isolated CD11b+ splenocytes were co-cultured with PMA-stimulated CD3+ cells in CFSE-based proliferation assay. Splenoctyes were labeled with CFSE as previously described. CFSE-labeled cells were co-cultured with BALF cells or isolated CD11b+ cells from GalCer-treated mice in 96-well plates for 3 days at 37°C in a 5% CO2 atmosphere. To induce proliferation, CFSE-labeled cells were stimulated with PMA. CFSE-labeled splenocytes were analyzed by flow cytometer.

### Statistics

Values are expressed as means ± standard errors of the mean (SEMs). Differences between experimental and control groups were analyzed by the Kruskal-Wallis test followed by Scheffe's F-test. The Wilcoxon's test of Kaplan-Meier plots was used to analyze differences in animal survival. Significance was established at *P* < 0.05.
